# Properties of the Impact of Vision Impairment and Night Vision Questionnaires Among People With Intermediate Age-Related Macular Degeneration

**DOI:** 10.1167/tvst.8.5.3

**Published:** 2019-09-11

**Authors:** Myra B. McGuinness, Robert P. Finger, Zhichao Wu, Chi D. Luu, Fred K. Chen, Jenifer J. Arnold, Usha Chakravarthy, Wilson J. Heriot, Jim Runciman, Robyn H. Guymer, for the LEAD Study Group

**Affiliations:** 1 Centre for Eye Research Australia, East Melbourne, Australia; 2 Department of Ophthalmology, University of Bonn, Bonn, Germany; 3 Ophthalmology, Department of Surgery, University of Melbourne, Melbourne, Australia; 4 Centre for Ophthalmology and Visual Science (incorporating Lions Eye Institute), The University of Western Australia, Crawley, Australia; Department of Ophthalmology, Royal Perth Hospital, Perth, Australia; 5 Marsden Eye Research, Sydney, Australia; 6 Belfast Health and Social Care Trust, Belfast, Northern Ireland; 7 Retinology Institute, Glen Iris, Australia; 8 Adelaide Eye and Retinal Centre, Adelaide, Australia

**Keywords:** age-related macular degeneration, night vision, patient-reported outcomes, vision-related quality of life, visual impairment

## Abstract

**Purpose:**

To explore the psychometric properties of the Impact of Vision Impairment (IVI-28) and Night Vision Questionnaires (NVQ-10) among people with intermediate age-related macular degeneration (iAMD).

**Methods:**

Baseline responses were collected from 288 participants (aged 50–88 years, 74% female) in the Laser intervention in Early stages of Age-related macular Degeneration (LEAD) study in Australia and Northern Ireland. Psychometric properties (discrimination, ordering of thresholds, person separation, item miss-fit, and differential item functioning according to sex) were explored using grouped rating scale and partial credit models. Spearman's correlation was estimated to assess the association with measures of visual function (mean mesopic microperimetric sensitivity, best-corrected visual acuity, low-luminance visual acuity, and low-luminance deficit). The psychometric properties were then explored following recalibration of the instruments.

**Results:**

In this homogenous population, ceiling effects caused by relatively high levels of functional vision were evident for both instruments. The IVI-28 and NVQ-10 displayed suboptimal discrimination between levels of functional vision in iAMD and poor targeting among people with iAMD. The correlation between ability scores and measures of visual function was mild. In general, the NVQ-10 showed superior psychometric properties to the IVI-28 among these participants. No significant improvement in reliability could be gained following recalibration.

**Conclusions:**

Both instruments were designed for populations with more severe visual loss and poorly discriminate in this cohort of iAMD.

**Translational Relevance:**

New instruments that can capture the subtle changes in functional vision that occur early in AMD are required to aid evaluation of emerging interventions for iAMD.

## Introduction

Patient-reported outcomes from studies of age-related macular degeneration (AMD) are increasingly used to assess the patient-relevance of changes in retinal structure and function, to measure disease progression, and to detect potential efficacy of new interventions.^1^-^4^ In addition to fulfilling regulatory requirements, investigation of the self-reported ability to perform vision-mediated activities has the potential to provide details regarding visual function that cannot be captured using clinical testing.^5^ Best-corrected visual acuity (BCVA), for example, is not a suitable marker of disease progression in early stages of AMD, as visual acuity is maintained until later in the disease process.^6^ Furthermore, by accurately describing vision-related quality of life, we can differentiate between visual function (e.g., psychophysical clinical measures of vision, such as visual acuity or parametric sensitivity) and self-reported functional vision, which describes a person's ability to perform activities that depend on vision, and is thus more relevant to handicap arising from vision loss.^7^ Here, the quality of life that is related to visual function is the construct (or latent trait) of interest and referred to as functional vision below.

The Impact of Vision Impairment (IVI-28) questionnaire has been validated among participants with visual impairment resulting from the later stages of AMD.^8^,^9^ However, this questionnaire has not been validated for use among people with subtle visual symptoms and a mild decline in function, such as is the case in intermediate AMD (iAMD; in this case defined as bilateral large drusen, or medium drusen with pigment).^10^ The Night Vision Questionnaire (NVQ-10) was designed for participants with a range of AMD phenotypes. It has been used to measure functional vision under low luminance and/or low-lighting conditions among people with iAMD despite no clear published description of its discriminatory properties to date.^2^,^11^,^12^

Therefore, the aim of this paper was to investigate the psychometric properties of the IVI-28 and NVQ-10 when completed by people with iAMD. We provided a glossary of terms relating to psychometric properties used in this paper in the supplementary material (Supplementary File S1).

## Methods

### Participants

Participants were recruited as part of the Laser intervention in Early stages of Age-related macular Degeneration (LEAD) study, an investigator-initiated, multicenter, double-masked, randomized sham-controlled clinical trial. The primary objective of the study was to investigate the efficacy of subthreshold nanosecond laser treatment in slowing progression from iAMD to the later stages of the disease.^13^

The trial was conducted at five Australian sites and one site in Northern Ireland. The coordinating center and sponsor was the Centre for Eye Research Australia (CERA) and the study is registered with the Australian New Zealand Clinical Trials Registry (ACTRN12612000704897) and clinicaltrials.gov (NCT01790802). This study was conducted according to the Declaration of Helsinki and the protocol was approved at all sites by local institutional review boards. All study participants provided written informed consent prior to being enrolled.

The full description of the LEAD study design and baseline participant characteristics have been published previously.^13^,^14^ In brief, eligible participants were 50 years or older and were required to have at least one druse more than 125 μm in diameter within 1500 μm from the fovea in each eye as determined via color fundus photographs. These criteria were chosen to select iAMD participants with a high risk of AMD progression.^13^ Individuals with neovascular AMD, detected on fundus fluorescein angiography, or drusen-associated atrophy detected on multimodal imaging were excluded. All participants were required to have BCVA of 20/40 or more in each eye and people with cataract of grade 2 or worse according to the World Health Organization Simplified Cataract Grading System were excluded.^15^

Participants were required to have completed both the NVQ-10 and IVI-28 at baseline to be included in this analysis.

### Participant Characteristics and Measures of Visual Function

Demographic data and ocular and systemic medical history were collected from each participant at baseline. BCVA was recorded as the number of letters correct on the Early Treatment of Diabetic Retinopathy Study chart at 4 m. Low-luminance visual acuity was measured as the number of letters read on the Early Treatment of Diabetic Retinopathy Study chart at 4 m with the 2.0-log unit neutral density Kodak Wratten filter (Kodak, Rochester, NY) in place. The low-luminance deficit was calculated by subtracting low-luminance visual acuity from BCVA. Microperimetric sensitivity was assessed via the Macular Integrity Assessment (MAIA) perimeter (CenterVue, Padua, Italy) using a 37-point macular test protocol. Lens status was assessed according to the World Health Organization Simplified Cataract Grading System. Baseline retinal biomarker quantification (assessment of drusen, pigmentary abnormalities, geographic atrophy, etc.) and AMD stage were assessed by trained retinal graders via multimodal imaging. The presence of reticular pseudodrusen (RPD) was determined via optical coherence tomography, near-infrared reflectance, fundus autofluorescence, and color fundus photographs, assessed by a senior grader and senior medical retinal clinician.^14^

### Questionnaires

Baseline questionnaires were completed prior to allocation to treatment group. Questions and response categories were read to participants by a trained study examiner who recorded responses.

The IVI-28 is a 28-item instrument that has previously been shown to possess three valid and reliable subscales, including reading and accessing information (items 1, 3, 5–9, 14, 15), mobility and independence (2, 4, 10–13, 16–20), and emotional well-being (items 21–28).^16^ There are four response categories for each item as seen in Supplementary Table S1 (all supplementary tables and figs. are in Supplementary File S2). Items 1 to 13 had an additional response category (‘Don't do it for other reasons'), which was treated as missing for the purposes of this analysis.

As seen in Supplementary Table S2, the first three items of the NVQ-10 have five graded response categories with additional categories (‘Stopped doing it for other reasons' and ‘Not currently driving') treated as missing. There are four response categories for the remaining seven items.

Responses from each questionnaire were reverse coded so that a score of zero was allocated for the lowest level of functional vision.

### Statistical Methods

Because instruments with differing numbers of response categories between items (such as the NVQ-10) cannot be assessed using a traditional rating scale model and partial credit models lack the invariance preferred when comparing scores longitudinally, grouped (hybrid) rating scale models were used to generate calibrated person ability scores for each participant and difficulty parameters for each item. Items from the IVI-28 and NVQ-10 were grouped according to their subscales as described above. In addition to assessing each instrument as a whole using grouped rating scale models, subscales were assessed independently using single-rating scale models.

The difference between mean item difficulty and mean person ability was used to assess the instruments' ability to target participants with iAMD. The number of functional vision strata distinguishable within this cohort was estimated via the real person separation coefficient (PSC; equal to the true population standard deviation divided by real root mean square error). The infit mean square (MNSQ) standardized residual value of each item was examined to assess the predictability of responses, and principal component analyses of model residuals were conducted to explore variation in model fit between items. Differences in responses to each item according to sex were assessed via estimation of differential item function contrasts (difference between differential item function measures of males and females). Partial credit models were generated to assess the rating scale model assumption of invariance of threshold steps between items.

Spearman's correlation coefficient was used to assess the association between calibrated person ability scores and each of BCVA, low-luminance visual acuity, low-luminance deficit, and microperimetric sensitivity (from eye with better function for that measure, values available for one eye only set to missing). Ability scores were compared between participants with and without RPD in either eye using the Wilcoxon rank sum test, as RPD are known to be associated with poor rod function, which is relevant to the functional impact assessed by the NVQ-10.^17^ The Wilcoxon rank sum test was also used to compared ability scores between participants who were pseudophakic and those with natural lenses.

Following assessment of psychometric properties, the instruments were iteratively recalibrated to order thresholds and improve model fit. First, response categories from subscales with evidence of disordered thresholds were collapsed. Next, items with high levels of misfit (MNSQ <0.7 or >1.3) were dropped if their omission did not reduce the ability of the instrument to discriminate between participants with different levels of functional vision (as measured via the PSC).^18^ This process was conducted for each instrument separately.

Item response theory models were generated and analyzed using Winsteps version 3.71.0.1 (Beaverton, OR) and the remaining analyses were conducted using Stata/SE version 15.1 (College Station, TX).

## Results

Of the 292 participants enrolled into LEAD and randomized between July 2012 and April 2015, 288 (99%) completed both questionnaires at baseline. Participants were aged between 50 and 89 years (mean 70, SD 7.6) and 212 (74%) were female. Baseline characteristics and measures of visual function can be seen in Table 1.

**Table 1:** Baseline Characteristics for Participants of the LEAD Study (n = 288), 2012–2018

	Distribution
Age at randomization, y, mean (SD)	70.1 (7.6)
Sex, *N* (%)	
Male	76 (26.4)
Female	212 (73.6)
Race, *N* (%)	
Caucasian	259 (89.9)
Other	29 (10.1)
Reticular pseudodrusen in either eye, *N* (%)	
Absent or questionable	213 (74.0)
Yes	75 (26.0)
Lens status, *N* (%)	
Both eyes natural lens	230 (79.9)
One eye pseudophakic	4 (1.4)
Both eyes pseudophakic	54 (18.8)
Visual function in best seeing eye	
MAIA mean sensitivity^a^, db, median [IQR]	27.1 [26.9, 28.0]
Best-corrected visual acuity (letters), mean (SD)	86.7 [20/20] (4.9)
Low-luminance visual acuity^a^ (letters), mean (SD)	73.5 [20/40] (6.2)
Low-luminance deficit^a^ (letters), mean (SD)	11.9 (4.0)

a Missing values for mean sensitivity, low-luminance visual acuity, and low-luminance deficit (*n* = 1 for each).

There was no evidence of participant fatigue for either instrument, with the majority of missing responses (and choice of response ‘Don't do it for other reasons') being recorded for earlier items rather than later items and no evidence of decreasing scores with increasing item number. The distribution of patient responses for both instruments was skewed toward higher functional vision for all items (Table 2). There was evidence of a ceiling effect for all items of both questionnaires, with the median response equal to the maximum category for all but two items.

**Table 2:** Item Characteristics of the IVI-28 and NVQ-10 in the LEAD Study (n = 288)

Item	Missing Responses^a^ (%)	Response Category				Infit Mean-Square^b^	Difficulty^b^	Discrimination^b^
		Median	Minimum	Maximum	Skew			
IVI-28								
1	0.3	3	0	3	−4.0	1.25	0.25	0.90
2	2.1	3	1	3	−4.7	0.90	−0.44	1.02
3	0.3	3	1	3	−4.3	1.05	−0.27	0.98
4	0.0	3	2	3	−16.9	1.01	−3.38	1.01
5	0.0	3	1	3	−4.6	1.55	−0.21	0.82
6	0.0	3	2	3	−4.8	1.01	−1.00	1.02
7	0.0	3	1	3	−4.0	1.24	−0.21	0.87
8	0.0	3	0	3	−2.1	1.00	1.60	0.95
9	0.0	3	1	3	−3.9	1.14	−0.32	0.94
10	0.0	3	0	3	−3.9	0.95	0.41	1.06
11	0.0	3	0	3	−2.7	0.90	1.36	1.00
12	0.7	3	0	3	−3.9	0.77	0.39	1.16
13	0.0	3	0	3	−2.4	0.93	1.38	0.99
14	0.0	3	0	3	−3.3	0.97	0.88	1.09
15	0.0	3	0	3	−3.8	1.00	0.36	1.09
16	0.0	3	1	3	−5.8	0.89	−0.71	1.13
17	0.0	3	1	3	−6.1	1.10	−0.80	1.04
18	0.0	3	1	3	−4.1	0.68	0.08	1.22
19	0.0	3	0	3	−4.8	1.01	0.04	0.96
20	0.0	3	0	3	−4.4	0.79	0.17	1.18
21	0.0	3	1	3	−4.8	0.94	−0.80	1.12
22	0.0	3	0	3	−2.3	0.98	1.39	1.06
23	0.0	3	1	3	−8.8	1.20	−2.01	1.04
24	0.0	3	0	3	−4.5	1.38	−0.22	0.88
25	0.0	3	0	3	−1.3	1.38	2.54	0.50
26	0.0	3	0	3	−4.0	1.76	0.31	0.66
27	0.0	3	1	3	−5.8	1.21	−1.13	1.02
28	0.0	3	0	3	−3.4	0.79	0.31	1.23
NVQ-10								
1	11.8	4	0	4	−2.5	1.09	−0.28	1.17
2	12.2	3	0	4	−1.6	0.80	0.67	1.08
3	12.5	4	0	4	−1.5	0.80	0.64	1.15
4	0.0	3	0	3	−1.6	1.19	0.14	0.93
5	0.0	3	0	3	−1.5	0.87	0.10	1.17
6	0.0	2	0	3	−0.6	0.85	1.58	1.02
7	0.0	3	0	3	−3.4	1.81	−1.51	0.73
8	0.0	3	0	3	−1.3	0.91	0.45	1.14
9	0.0	3	0	3	−1.3	1.08	0.59	0.93
10	0.0	3	0	3	−4.8	1.97	−2.37	0.76

a The response category ‘Don't do it for other reasons' for IVI-28 items 1–13 and the response categories ‘Stopped doing this for other reasons' and ‘Not currently driving' for NVQ-10 items 1–3 were treated as missing for the purposes of this analysis.

b Model parameters were estimated using grouped rating scale models. Item difficulty describes the level of functional vision required to perform the task of interest and item discrimination describes how well response categories correlate with levels of functional vision measured by person ability scores.

### IVI-28

Item 2 (recreational activities) had the most missing responses (*n* = 6/288, 2.1%). No participants indicated that vision had impacted their thoughts/activities “a lot” for 13 of the 28 items and very few participants (≤2.4%) chose this response for the remaining items (see Supplementary Table S1).

The grouped rating scale model explained only 46% of the variance in the data, showed poor discrimination (PSC 0.8, implying participants in this cohort could not be classified into at least two separate strata of functional vision levels) and very poor targeting of the cohort of interest (4.8 logits difference between mean item difficulty and mean person ability; Fig. and Table 3). Step difficulties advanced by less than 1.4 logits for each of the response categories (except for the final step in the emotional subscale) indicating that combining response categories may be beneficial (see Supplementary Table S3).^19^

**Figure:**
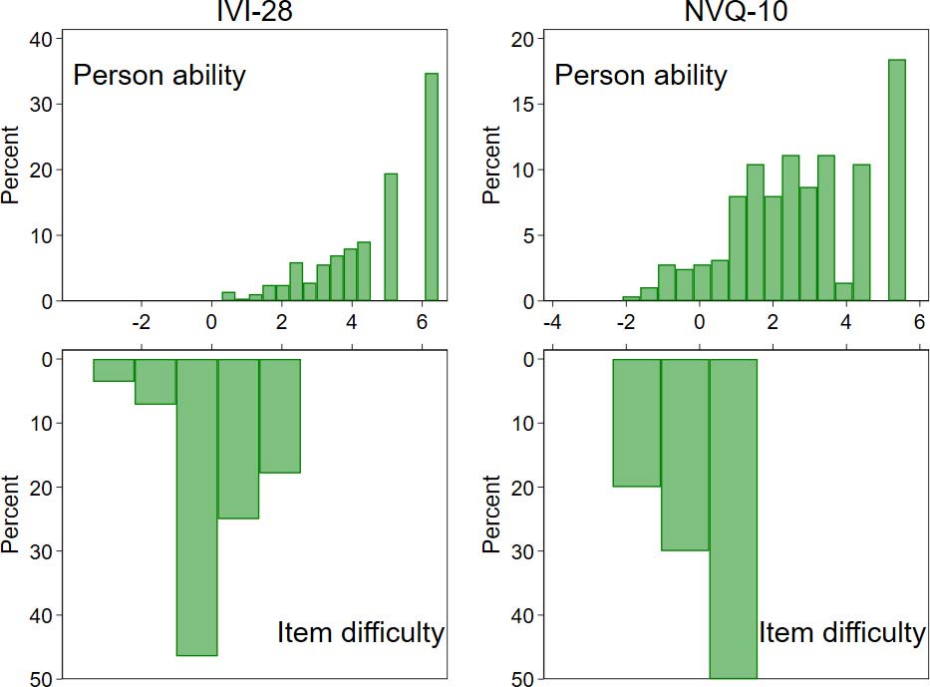
Person-item map for the IVI-28 and the NVQ-10 from the LEAD Study. Person ability and item difficulty measured in logits.

**Table 3:** Summary Psychometric Properties for the IVI-28 and NVQ-10 from the LEAD Study

	IVI-28 Items^a^				NVQ-10 Items^a^		
	All	R	M	E	All	C	O
Number of items	28	9	11	8	10	3	7
Number of miss-fitting items^b^ (mean-square standardized residual <0.7 or >1.3)	4	1	1	2	2	0	2
Number of items with differential functioning by sex^b^ (difference in measures >1)	7	5	5	1	0	0	1
Raw variance explained by measures^b^ (%)	**46**	**34**	**35**	**48**	**58**	62	62
Unexplained variance in first contrast^b^ (Eigenvalue units)^b^	**2.3**	1.6	1.6	2.0	**2.2**	1.7	**2.5**
Targeting^b^ (logits) (difference between mean item difficulty and mean person ability)	**4.7**	**4.8**	**4.5**	**4.9**	**2.8**	**3.5**	**1.7**
Person separation coefficient^b^ (real mean-square error/true SD)	**0.8**	**0.0**	**0.0**	**0.0**	1.5	1.1	2.1
Strata of functional vision	**1.4**	**0.3**	**0.3**	**0.3**	2.4	1.8	3.1

a Subscales: (R) reading and accessing information, (M) mobility and independence, (E) emotional well-being, (C) car travel, (O) other.

b Assessed using a grouped rating scale model for all items and a rating scale model for subscales.

Values considered to be suboptimal (raw variance <50%, unexplained variance >2.0 Eigenvalue units, targeting >1.0 logit, person separation coefficient <0.8, strata <2) are shown in boldface type.

Eleven of the items showed poor discrimination (<1) and the departure from expected response was high for items 5 and 24-26 as demonstrated by high MNSQ values (≥1.38, Table 2). Principal component analysis revealed unidimensionality within each of the three subscales (unexplained variance in first contrast 1.6–2.0 eigenvalue units, Table 3). However, when all items were assessed in a grouped rating scale model there was a slight departure from unidimensionality (2.3 eigenvalue units). PCA revealed several items in the emotional wellbeing subscale had contrast loadings and item difficulty values which were clustered apart from those of the remaining items, suggesting that the emotional subscale may reflect a different latent trait to the remaining items.

There was evidence of differential function between males and females (Supplementary Fig. S1 and Table 1). Noticeably, item 4 (visiting friends and family) was, on average, more difficult for males, and item 6 (looking after appearance) was more difficult for females.

### NVQ-10

Items 1 through 3 (which relate to driving) were treated as missing for 11.8%, 12.2%, and 12.5% participants respectively (Table 2). Females were overrepresented in this group (94%).

After fitting a grouped rating scale model, the step thresholds for the first subscale were found to be disordered (meaning that the probability of choosing each response category did not increase monotonically with person ability, see Supplementary Table S4). This is consistent with the frequency of responses seen in Supplementary Table S2, which shows that more participants chose the category corresponding to the lowest level of the trait (‘Stopped doing this because of your eyesight') than the second lowest level (‘Extreme difficulty', lighter shading indicates lower frequency of responses and implies disordered thresholds).

Items 4, 7, 9, and 10 showed poor discrimination (<1) and items 7 and 10 displayed highly variable responses after adjusting for person ability scores (infit MNSQ 1.8 and 2.0, respectively, Table 2). The model displayed poor targeting of the cohort of interest, with a difference of 2.4 logits between the mean person ability and mean item difficulty (Fig.). The PSC was 1.5 indicating the instrument could discriminate at least two separate strata of functional vision among this cohort. The grouped rating scale model explained 58% of variance in the data and principal component analysis revealed a contrast in patterns among the residuals (unexplained variance in first contrast = 2.2 eigenvalue units), dividing items 1 through 4 (relating to car travel and driving) from the rest.

There was no evidence of substantial differential item functioning items across sex (Supplementary Fig. S2).

### Estimated Levels of Functional Vision

The estimated ability scores derived from the IVI-28 ranged from 0.3 (greater impact of vision impairment) to 6.5 (better functional vision) with a median of 5.2 (interquartile range [IQR] 3.6–6.5). The estimated NVQ-10 ability scores ranged from −2.1 to 5.6 with a median of 2.7 (IQR 1.6–4.3).

There was a very weak correlation between the IVI-28 and NVQ-10 ability scores and each of BCVA (*ρ* = 0.12 and 0.16), low-luminance visual acuity (*ρ* = 0.15 and 0.21), and mean microperimetric sensitivity (*ρ* = 0.12 and 0.14, respectively), and a negligible correlation between the ability scores and low-luminance deficit (*ρ* = −0.08 and −0.11) as seen in Supplementary Figures S3 and S4 *(P* < 0.05 for each except low luminance deficit: IVI-*28 P* = 0.167, NVQ-28 *P* = 0.063). There was no evidence of a difference in ability scores between participants who did and did not have RPD in either eye (IVI-28 *P* = 0.56, NVQ-10 *P* = 0.82), nor between those who pseudophakic bilaterally and those who had natural lenses in both eyes (IVI-28 *P* = 0.98, NVQ-10 *P* = 0.62).

### Recalibration of Instruments

After fitting a partial credit model, disordered thresholds were evident for 11 of the 28 IVI-28 items (i.e., a decrease in person ability scores corresponding to a one-step increase in response category as suggested by the shading of cells in Supplementary Table S3). Therefore, the first two response categories of each item were collapsed, resulting in a minimal improvement in PSC and targeting. Removal of the emotional well-being subscale greatly decreased the discrimination of the instrument (PSC 0.2) while the results of principal components analysis still indicated multidimensionality (unexplained variance in first contrast = 2.1 eigenvalue units).

The first two response categories from NVQ-10 items 1 through 3 were collapsed to create four categories to order step thresholds, aligning the number of response categories with the remaining items. Items were regrouped into thematic subscales of items 1 through 4 (relating to travel) and items 5 through 10 (nontravel related). Then, items 7 and 10, which consistently displayed high infit values, were dropped. Following these modifications, neither discrimination nor targeting improved and dimensionality improved only slightly (2.1 eigenvalue units).

## Discussion

In this study of participants recruited with iAMD and baseline BVCA of 20/40 or better, we found a narrow range of responses to the current version of the IVI-28 which correspond to higher levels of functional vision. The IVI-28 was developed for use in cohorts, which include people with more severe visual loss, and the ceiling effects observed in this study indicate that it would be more appropriate for cohorts with a greater range of functional vision. Given the strict eligibility criteria with respect to stage of AMD and the BCVA requirements in the present study (chosen to select individuals with a high risk of progressing from iAMD to the later stages of AMD) the absence of heterogeneity in responses among the LEAD study participants is not surprising.

### Comparison to Other Research

Results from the Alabama Study on Early Age-Related Macular Degeneration suggest that, among people without severe visual impairment, instruments relating to activities performed in mesopic conditions are more likely to detect a decline in vision-related quality of life over 3 years compared with those relating to daytime activities.^20^ These results, along with our findings of marginally superior targeting and discrimination of the NVQ-10 compared with the IVI-28, provide support for the use of low-luminance questionnaires for the detection of early deficits in functional vision.

A review of 48 ophthalmic questionnaires was published in 2013.^21^ At that time the authors recommended the IVI-28 for assessing patient reported outcomes among people with macular diseases. In contrast to the findings of our analysis, the IVI has been shown to be valid and reliable among participants with more severe vision impairment, including those with late AMD. Fenwick and co-authors^22^ reported that good targeting could be maintained after dropping several items with substantial missing data. Similar to analyses conducted among participants with multiple causes of low vision, we found evidence of differential factor loadings between the items of emotional and wellbeing subscale and the remaining items.^8^ Despite this, Goldstein and co-authors^23^ opined that a summary IVI-28 score representing items from all subscales, which was generated using their large sample−derived parameters would be adequate when reporting outcomes from medical interventions.

We have previously reported a similar range of NVQ-10 ability scores among participants with iAMD.^11^ The psychometric properties of other instruments designed to capture functional vision in low luminance have thus far only been described in detail among groups, which included participants with more severe vision loss.^24^–^26^ In the above-mentioned 2013 review, the authors did not specifically assess instruments for their ability to capture dysfunction in the presence of mild visual impairment.^21^ However, it is possible that some of the items from questionnaires designed for other conditions, such as refractive error, cataract and glaucoma may be suitable for use among people with iAMD.^27^–^29^

### Strengths and Limitations

The full spectrum of iAMD phenotypes was not represented among the participants of this study. In this homogenous cohort, the extreme response option, which represented poor functional vision was never selected by any participant for almost half of the IVI-28 items at baseline. In addition, fewer than half of the NVQ-10 items had 10 or more participants respond to each category, as recommended for stable estimation of step thresholds.^19^ Collapsing response categories improved model parameters in this cohort; however, this may result in the inability to detect subtle changes as AMD progresses.

Strengths include the large sample size and the prospective study design, which enabled standardized testing of all participants. Comprehensive assessment of visual function allowed comparisons to be made between instrument scores and test performance in mesopic conditions.

### Biological Plausibility

We observed some evidence of criterion-related validity as there was a weak correlation between each of the instruments and low-luminance visual acuity. Despite relatively good BCVA, people with iAMD have been shown to have poorer visual function under mesopic conditions.^6^,^30^ Anecdotally, patients with iAMD often report a prolonged period of adjustment when moving between areas of high and low luminance and when waking from sleep. These findings have chiefly been attributed to impairment in recovery of rod photoreceptor function. The presence of RPD prior to the development of AMD has also been correlated with decreased visual function in scotopic and mesopic conditions.^31^ Therefore, we hypothesized that instruments that probe aspects of functional ability in low-light settings may have a greater capacity to detect the earliest changes in retinal structure associated with AMD. However, no differences in instrument scores emerged based on RPD status in this cohort.

### Further Research

Although the current instruments are capable of detecting decline in vision-related quality of life associated with progression from early to late stage AMD,^32^ new instruments that can capture the subtle changes in functional vision that occur prior to the development of atrophy and neovascularization are required to assess the safety and efficacy of emerging interventions. This is particularly important for interventions, which are intended to slow AMD progression rather than improve vision. The MACUSTAR consortium are currently designing an instrument to suit these needs.^10^ New instruments should be designed to include items related to activities that are conducted commonly in low levels of luminance and items that capture the rate of dark and light adaptation. The precision of the NVQ-10 estimates was limited by the number of people who did not drive a car. Given the number of people in this population who do not drive for reasons other than vision, less prominence may need to be given to questions relating to driving. However, the identification of items that can capture the ability to perform tasks, which are as visually demanding as driving is likely to prove challenging.

Differences in responses between men and women of the same overall functional ability were detected for seven of the IVI-28 items. The perceived complexity of activities relating to personal appearance and social habits are likely to differ between the sexes and these differences should be considered when developing future instruments and when comparing results between cohorts with differing demographics.

Ideally, new instruments should be designed to fit a rating scale–type model with the favorable property of invariance, which is particularly desirable when comparing responses across time-points and cohorts. Among our cohort, in which a somewhat narrow range of visual function was demonstrated, decreasing levels of functional ability did not correspond to decreasing probabilities of a respondent being observed in lower response categories; this is an essential feature of the rating scale model.^19^ Given the poor fit of our data to a rating scale model, models with greater flexibility, such as partial credit models, may be more appropriate, especially when making inferences about functional vision at a single timepoint.^33^ However, these models make it more difficult to compare results between cohorts. When available, standardized calibration parameters developed using a large sample, such as those developed for the IVI-28 and published by Goldstein and co-authors,^23^ should be used to allow direct comparison between studies.

The questionnaire data presented in this paper will be analyzed to investigate the natural history of functional vision among participants of the LEAD study over a 3-year period and to investigate the association between functional vision and progression to late AMD.

### Conclusions

Neither instrument performed optimally in this homogenous sample of participants with good visual acuity. Thus, a new instrument needs to be developed which specifically addresses the problems encountered early in the AMD disease process and allows for more precise assessment of patient reported outcomes within studies of interventions that aim to slow the progression of iAMD.

## Supplementary Material

Supplement 1

Supplement 2
