# Supplementary material for: Properties of the Impact of Vision Impairment and Night Vision Questionnaires Among People With Intermediate Age-Related Macular Degeneration
Source: Transl Vis Sci Technol. 2019 Sep 11;8(5):3. doi: 10.1167/tvst.8.5.3 (PMC6753972; doi:10.1167/tvst.8.5.3)
Supplement: Supplement 1 [file tvst-08-04-25_s01.pdf]

Supplementary Material A: Glossary

Properties of the Impact of Vision Impairment and Night Vision Questionnaires among people with intermediate age-related macular degeneration

Term	Description
Differential item functioning	Differential item functioning (DIF) occurs if responses to an item differ systematically among people with the same overall ability score. For example, an item might display DIF if ethnicity impacts on the interpretation of the perceived difficulty of the visual task addressed in that item. The difference in the differential item functions between categories of a given characteristic (such as ethnicity, gender or cause of vision loss) is known as the contrast and is likely to be <1 in the absence of DIF.
Dimensionality	This refers to the number of latent traits that an instrument encapsulates. Ideally, an instrument should be unidimensional, meaning that the total score reflects only one underling and pre-specified concept or latent trait.
Infit	This statistic measures the fit of a statistical model to participant responses using weighted standardised residuals. In general, infit mean square (MNSQ) values <0.7 indicate an *over-fitted* model for an item, and that the item may be redundant given the data obtained from other items (i.e. it is highly correlated with another item). Items with MNSQ values >1.3 indicate an *under-fitted* model for that item, meaning there is considerable variation in responses at given person ability scores, and that the item may be measuring a different latent trait to the other items.
Instrument	This is another name for a questionnaire or scale. The NVQ-10 and the IVI-28 are examples of instruments.
Invariance	Invariance refers to whether model parameters change between items, instruments or participants.<br><br>*Item invariance* An item is said to have invariance when the difficulty parameter for that item remains the same across different instruments which are intended to measure the same latent trait.<br><br>*Group invariance* describes whether the item parameters from a model remain the same regardless of the abilities of the people completing the questionnaires.<br><br>*Step invariance* refers to items in a scale which have the same distance between step thresholds of adjacent categories.

Term	Description
Item	Each question of an instrument is referred to as an item. The NVQ-10, for example, has ten items.
Item difficulty	This is a measure of the level of the latent trait that is required to respond positively to each item. Item difficulty is measured in logits. If the subject of an item is considered to be difficult (such as driving in the rain at night) the item difficulty will be greater than zero.
Item discrimination	This reveals how well item responses correlate to overall person ability scores, i.e. whether people with the highest level of ability choose the most difficult response option. Item discrimination should be close to one. Items with discrimination of <1 do not measure the latent trait well.
Latent trait	Also referred to as a construct or latent variable. It refers to a trait which cannot be measured directly, such as vision-related quality of life or impact of vision on ability to perform activities of daily living.
Logit	The logit is a unit of measurement which describes the level of a latent trait. It can be thought of as the logarithm of the odds of selecting increasingly difficult response categories. When calibrating an instrument, the average item difficulty is set to zero logits.
Model	A model is a statistical algorithm that is used to estimate a person's ability or level of a latent trait using item responses.<br><br>*Rating scale models* are used to analyse data from instruments with polytomous items (meaning each question has more than two ordered response categories). The same step threshold parameters are applied to every item and therefore each item needs to have the same number of response categories. It is assumed that the change in difficulty represented by a one-step difference in response categories (e.g., the difference between *somewhat bothered* and *very bothered*) is the same for every item, i.e., that the items display step invariance.<br><br>*Grouped rating scale models* are made up of two or more rating scale models. For example, the NVQ-10 has two sets of response categories: the first set has six response categories and relates to how difficult tasks are to perform, the second set has four response categories and relates to how bothered people are by their vision. Therefore, different sets of step threshold parameters are needed for each sub-scale.<br><br>*Partial credit models* use different step threshold parameters for each item. These models are used when participant responses do not meet the statistical assumptions of rating scale models.
Person ability	A person's ability refers to the level of the latent trait that they possess. It is estimated using models and measured in logits.

Term	Description
Principal component analysis	This is an analysis of the residual values generated from a statistical model. It is used to group correlated items and explore the dimensionality of an instrument.<br>*Contrasts* are dimensions formed by groups of items.<br>*Eigenvalue* This measures the strength of the contrasts and can be interpreted as the number of items in each contrast. If the first contrast has unexplained variance of ≥2 eigenvalue units, the instrument may be considered to be multidimensional.<br>*Contrast loadings* for each item can be positive or negative. The greater the magnitude of the loadings, the more variation across the instrument. Factor loadings >|0.4| are considered to be high.<br>*Raw variance* This is the variation between participant responses. Ideally the model will explain >50% of the raw variance.
Real person separation coefficient	This is a measure of scale precision. It is estimated by dividing the population standard deviation by the real root mean square error (RMSE, the average of the standard errors). This value is used to assess how well a questionnaire can differentiate people into groups (or strata) defined by their abilities using the formula: $Person\ strata = \frac{(4 \times RPSC + 1)}{3}$. Ideally, the RPSC should be ≥2, which indicates that the questionnaire can differentiate between people with low, medium and high levels of ability.
Step thresholds	Step thresholds (measured in logits) describe the ability level at which two adjacent response categories have an equal probability of being chosen. Step thresholds should increase monotonically (i.e. response categories should be ordered from easiest to hardest) and are otherwise described as disordered.
Targeting	Targeting refers to how well the difficulty of the items in an instrument reflect the abilities of the population of interest. It is measured in logits as the difference between mean item difficulty (set to zero) and mean person ability. An instrument with <1 logit difference is considered to target the population well. Targeting can be visually inspected via person-item maps which compare the distribution of item difficulties and person ability scores. An instrument which is targeted toward people with severe vision impairment, for example, may be too “easy” for people with mild vision impairment.

IVI-28 = 28-item Impact of Vision Impairment questionnaire, NVQ-10 = 10 item Night Vision Questionnaire.
