# Supplementary material for: Properties of the Impact of Vision Impairment and Night Vision Questionnaires Among People With Intermediate Age-Related Macular Degeneration
Source: Transl Vis Sci Technol. 2019 Sep 11;8(5):3. doi: 10.1167/tvst.8.5.3 (PMC6753972; doi:10.1167/tvst.8.5.3)
Supplement: Supplement 2 [file tvst-08-04-25_s02.pdf]

# Supplementary Material B

**Properties of the Impact of Vision Impairment and Night Vision Questionnaires among people with intermediate age-related macular degeneration**

## LEAD Study Group

### Data safety monitoring committee

S Al-Qureshi (chair), L Busija, I Constable

### Medical monitor

D Louis

### Endpoint adjudicating committee

C Harper, S Wickremasinghe, P Van Wijngaarden, L Lim

### Australian sites

**Adelaide Eye and Retina Centre**

S Durkin (principal investigator), J Runciman, J Gihotra, J Muecke, K Haywood, C Brko, J Paley, M Smith, C Luscombe, R Vincent, and D Lee

**Centre for Eye Research Australia, University of Melbourne**

RH Guymer (principal investigator), C Luu, Z Wu, LAB Hodgson, K Brassington, E Caruso, M McGuinness, N Tindill, KZ Aung, E Baglin, P Sharangan, CA Harper, S Wickremasinghe, L Lim, P Van Wijngaarden, S Sandhu, T Nguyen, A Cohn, D Qatarneh, L Robman, G Makeyeva, R Tan, S Taori, K Creese, M Chen, D Ong, S No, R Kandasamy, SW Lim, M Okada, D Cugley, R O'Day, P Keller, K Lee, E Alessandrello, J Alessi-Calandro, M Kolic, T Wu, S Griffin, JJ Lek

**Retinology Institute Victoria, Melbourne**

W Heriot (principal investigator), X Fagan, R McIntosh, C Lowe, J Boyle, and O Shanahan

**Lions Eye Institute, Perth**

FK Chen (principal investigator), IL McAllister, TW Isaacs, A Shaw, C Balarantnasingam, Y Chen, W Cunningham, R Viljoen, K Kennelly, R Blum, S Arunachalam, H Razavi, M Adams, R McKeone, T Busby, G Lingham, R Matthews, H Brown, J Bryant, R Cowles, S Radtke, C Barry, E Wong, F Shilton, A Soloshenko, A Jason, A Lin, A McSweeney, A King, B Shalan, D Xie, H Vu, I Tang, K Mather, M Cuypers, and M Cheng

**Marsden Eye Research, Sydney**

J Arnold (principal investigator) A Luckie, D Chan, J Chang, T Tan, L Koh, H Cass, R Fitzsimons, T Forsyth, A Nguyen, V Ghebrial, H Ayson, A Graham, and M Firibaldi

## International site

**Belfast Health and Social Care Trust, Northern Ireland**

U Chakravarthy (principal investigator), L Kelly, K Gillvray, M Williams, G Casalino, G Mangoris, R Das, T Peto, L Toth, M Quinn, R Denham, NJ Lavery, G Sterrett, V Silvestri, G Young, K Graham, J Keenan, L Doyle, T Douglas, D Burns, P Wright, L Scullion

**Supplementary Table S1**: Percentage of responses for each category of the Impact of Vision Impairment questionnaire in the Laser intervention in Early Age-related macular Degeneration (LEAD) Study (n = 288).

| Item | Question | 0<br>A lot | 1<br>A fair amount | 2<br>A little | 3<br>Not at all | Don't do it for other reasons* |
|---|---|---|---|---|---|---|
| **In the PAST MONTH, how much has YOUR SIGHT INTERFERRED with the following activities:** | | | | | | |
| 1 | Your ability to see and enjoy T.V.? | 0.3 | 1.0 | 8.0 | 90.3 | 0.3 |
| 2 | Taking part in recreational activities, such as bowling, walking or golf? | 0.0 | 0.3 | 4.5 | 93.1 | 2.1 |
| 3 | Shopping? (finding what you want and paying for it) | 0.0 | 0.7 | 5.6 | 93.4 | 0.3 |
| 4 | Visiting friends and family? | 0.0 | 0.0 | 0.3 | 99.7 | 0.0 |
| 5 | Recognising and meeting people? | 0.0 | 1.7 | 4.2 | 94.1 | 0.0 |
| 6 | Generally looking after your appearance? (face, hair, clothing, etc) | 0.0 | 0.0 | 3.8 | 96.2 | 0.0 |
| 7 | Opening packaging? (for example, around food, medicines) | 0.0 | 0.7 | 6.3 | 93.1 | 0.0 |
| 8 | Reading labels or instructions on medicines? | 1.0 | 4.2 | 18.4 | 76.4 | 0.0 |
| 9 | Operating household appliances and the telephone? | 0.0 | 0.3 | 6.3 | 93.4 | 0.0 |
| 10 | How much has your eyesight interfered with getting about outdoors? (on the pavement or crossing the street)? | 0.3 | 1.7 | 7.3 | 90.6 | 0.0 |
| 11 | In the past month, how often has your eyesight made you go carefully to avoid falling or tripping? | 1.4 | 2.1 | 16.0 | 80.6 | 0.0 |
| 12 | In general, how much has your eyesight interfered with travelling or using transport? | 0.3 | 1.4 | 7.6 | 89.9 | 0.7 |
| 13 | Going down steps, stairs, or curbs? | 0.7 | 3.5 | 15.6 | 80.2 | 0.0 |
| 14 | Reading ordinary size print? (for example, newspaper) | 1.0 | 1.7 | 11.5 | 85.8 | 0.0 |
| 15 | Getting information that you need? | 0.3 | 2.1 | 6.9 | 90.6 | 0.0 |
| **In the PAST MONTH, how often has YOUR SIGHT MADE YOU CONCERNED OR WORRIED about the following:** | | | | | | |
| 16 | Your general safety at home? | 0.0 | 0.7 | 3.1 | 96.2 | 0.0 |
| 17 | Spilling or breaking things? | 0.0 | 0.7 | 2.8 | 96.5 | 0.0 |
| 18 | Your general safety when out of your home? | 0.0 | 1.7 | 5.6 | 92.7 | 0.0 |
| 19 | In the past month, how often has your eyesight stopped you doing things you want to do? | 0.3 | 1.0 | 5.6 | 93.1 | 0.0 |
| 20 | In the past month, how often have you needed help from other people because of your eyesight? | 0.3 | 1.0 | 6.6 | 92.0 | 0.0 |

| Item | Question | 0<br>A lot | 1<br>A fair amount | 2<br>A little | 3<br>Not at all | Don't do it for other reasons* |
|---|---|---|---|---|---|---|
| 21 | Have you felt embarrassed because of your eyesight? | 0.0 | 0.7 | 4.5 | 94.8 | 0.0 |
| 22 | Have you felt frustrated or annoyed because of your eyesight? | 1.4 | 3.5 | 17.4 | 77.8 | 0.0 |
| 23 | Have you felt lonely or isolated because of your eyesight? | 0.0 | 0.3 | 1.4 | 98.3 | 0.0 |
| 24 | Have you felt sad or low because of your eyesight? | 0.3 | 0.7 | 6.9 | 92.0 | 0.0 |
| 25 | In the past month, how often has your eyesight made you concerned or worried about your eyesight getting worse? | 2.4 | 5.9 | 34.7 | 56.9 | 0.0 |
| 26 | In the past month, how often have you worried about coping with everyday life? | 1.0 | 0.3 | 10.1 | 88.5 | 0.0 |
| 27 | Have you felt like a nuisance or a burden because of your eyesight? | 0.0 | 0.7 | 3.1 | 96.2 | 0.0 |
| 28 | In the past month, how much has your eyesight interfered with your life in general? | 0.3 | 1.7 | 9.4 | 88.5 | 0.0 |

* This response was treated as missing for the purposes of this analysis
Darker cells indicate more responses
Empty cells indicate that the response was not available for that item

**Supplementary Table S2:** Percentage of responses for each category of the Night Vision Questionnaire in the Laser intervention in Early Age-related macular Degeneration (LEAD) Study (n = 288)

| Item | Question | 0 Stopped doing this because of your eyesight | 1 Extreme difficulty | 2 Moderate difficulty | 3 A little difficulty | 4 No difficulty at all | Stopped doing this for other reasons* | Not currently driving* |
|---|---|---|---|---|---|---|---|---|
| 1 | How difficult is it for you to see moving objects, such as people or other cars when driving at night? Would you say you have: | 3.5 | 0.3 | 4.9 | 14.6 | 64.9 | 7.3 | 4.5 |
| 2 | How difficult do oncoming headlights or streetlights make it for you to drive at night? Would you say you have: | 3.5 | 2.1 | 9.0 | 29.5 | 43.8 | 7.6 | 4.5 |
| 3 | How difficult is it for you to read street signs when driving at night? Would you say you have: | 3.8 | 3.5 | 10.1 | 20.5 | 49.7 | 8.0 | 4.5 |
| 4 | How difficult is it for you to see street signs when you are a passenger in the car at night? Would you say you have: | NA | 3.8 | 8.0 | 23.3 | 64.9 | | |
| | **Tell us how bothered you are by these items** | **0 Very bothered** | **1 Somewhat bothered** | **2 A little bothered** | **3 Not bothered at all** | | | |
| 5 | Poor vision at night | 2.8 | 6.3 | 28.8 | 62.2 | | | |
| 6 | Problems in reading in dim light | 4.5 | 19.1 | 44.1 | 32.3 | | | |
| 7 | A dark spot in the middle of my vision in dim light | 2.1 | 2.1 | 8.7 | 87.2 | | | |
| 8 | Poor vision in dim lighting | 3.1 | 9.4 | 30.9 | 56.6 | | | |
| 9 | Problems adjusting to the dark when entering a theatre | 4.9 | 9.7 | 29.2 | 56.3 | | | |
| 10 | Trouble seeing the stars in the sky at night | 1.0 | 1.4 | 4.5 | 93.1 | | | |

* This response was treated as missing for the purposes of this analysis
Darker cells indicate more responses
Empty cells indicate that the response was not available for that item

**Supplementary Table S3**: Step thresholds (in logits) for the Impact of Visual Impairment (IVI-28) questionnaire from the Laser intervention in Early Age-related macular Degeneration (LEAD) Study.

| Model | Item | Step parameters | | | | |
|---|---|---|---|---|---|---|
| | | 1 | 2 | | 3 | |
| | | Threshold* | (Difference) | Threshold* | (Difference) | Threshold |
| *Subscale 1: Reading and accessing information* | | | | | | |
| Rating scale | | -1.05 | (0.90) | -0.15 | (1.35) | 1.20 |
| Partial credit | 1 | -0.36 | (-0.16) | -0.52 | (1.40) | 0.88 |
| | 3 | | | -0.52 | (1.04) | 0.52 |
| | 5 | | | 0.23 | (-0.46) | -0.23 |
| | 6 | | | 0 | (0) | 0 |
| | 7 | | | -0.66 | (1.32) | 0.66 |
| | 8 | -1.17 | (0.82) | -0.35 | (1.88) | 1.53 |
| | 9 | | | -1.01 | (2.02) | 1.01 |
| | 14 | -0.17 | (-0.56) | -0.73 | (1.63) | 0.90 |
| | 15 | -1.04 | (1.37) | 0.33 | (0.39) | 0.72 |
| *Subscale 2: Mobility and independence* | | | | | | |
| Rating scale | | -0.96 | (0.84) | -0.12 | (1.20) | 1.08 |
| Partial credit | 2 | | | -0.66 | (1.32) | 0.66 |
| | 4 | | | 0 | (0) | 0 |
| | 10 | -0.86 | (0.95) | 0.09 | (0.68) | 0.77 |
| | 11 | -0.23 | (-0.76) | -0.99 | (2.21) | 1.22 |
| | 12 | -0.66 | (0.47) | -0.19 | (1.04) | 0.85 |
| | 13 | -1.22 | (1.00) | -0.22 | (1.67) | 1.45 |
| | 16 | | | 0.06 | (-0.12) | -0.06 |
| | 17 | | | 0.18 | (-0.36) | -0.18 |
| | 18 | | | -0.07 | (0.14) | 0.07 |
| | 19 | -0.32 | (0.17) | -0.15 | (0.63) | 0.48 |
| | 20 | -0.34 | (0.01) | -0.33 | (1.00) | 0.67 |
| *Subscale 3: Emotional well-being* | | | | | | |
| Rating scale | | -1.00 | (0.41) | -0.59 | (2.19) | 1.60 |
| Partial credit | 21 | | | -0.32 | (0.64) | 0.32 |
| | 22 | -0.77 | (0.20) | -0.57 | (1.91) | 1.34 |
| | 23 | | | 0.52 | (-1.04) | -0.52 |
| | 24 | 0.06 | (-0.85) | -0.79 | (1.52) | 0.73 |
| | 25 | -1.26 | (0.19) | -1.07 | (3.40) | 2.33 |
| | 26 | 1.46 | (-3.68) | -2.22 | (2.98) | 0.76 |
| | 27 | | | 0.06 | (-0.12) | -0.06 |
| | 28 | -0.89 | (0.72) | -0.17 | (1.23) | 1.06 |

*Missing threshold values occur when no participant chooses the specified response category. Step thresholds identify the difficulty level (in logits) at which two adjacent response categories are equally likely to be chosen by a person of the corresponding ability level.
Shaded cells indicate a decrease in ability between response categories and disordered thresholds.

**Supplementary Table S4**: Step thresholds (in logits) for the Night Vision Questionnaire (NVQ-10) from the Laser intervention in Early Age-related macular Degeneration (LEAD) Study.

| **Model** | **Item** | **Step parameters** | | | | | | |
|---|---|---|---|---|---|---|---|---|
| | | **1** | **2** | | **3** | | **4** | |
| | | **Threshold** | **(Difference)** | **Threshold** | **(Difference)** | **Threshold** | **(Difference)** | **Threshold** |
| *Subscale 1: Driving* | | | | | | | | |
| Rating scale | | -0.24 | (-1.17) | -1.41 | (1.43) | 0.02 | (1.61) | 1.63 |
| Partial credit | 1 | 1.87 | (-4.45) | -2.58 | (2.37) | -0.21 | (1.14) | 0.93 |
| | 2 | -0.31 | (-1.29) | -1.60 | (1.29) | -0.31 | (2.53) | 2.22 |
| | 3 | -0.62 | (-0.48) | -1.10 | (1.27) | 0.17 | (1.38) | 1.55 |
| *Subscale 2: Other* | | | | | | | | |
| Rating scale | | -1.47 | (1.17) | -0.30 | (2.07) | 1.77 | | |
| Partial credit | 4 | -0.99 | (0.65) | -0.34 | (1.67) | 1.33 | | |
| | 5 | -1.02 | (0.28) | -0.74 | (2.50) | 1.76 | | |
| | 6 | -2.41 | (1.91) | -0.50 | (3.42) | 2.92 | | |
| | 7 | 0.24 | (-0.72) | -0.48 | (0.72) | 0.24 | | |
| | 8 | -1.43 | (0.97) | -0.46 | (2.35) | 1.89 | | |
| | 9 | -1.14 | (0.64) | -0.50 | (2.14) | 1.64 | | |
| | 10 | 0.26 | (-0.27) | -0.01 | (-0.23) | -0.24 | | |

Shaded cells indicate a decrease in ability between response categories and disordered thresholds

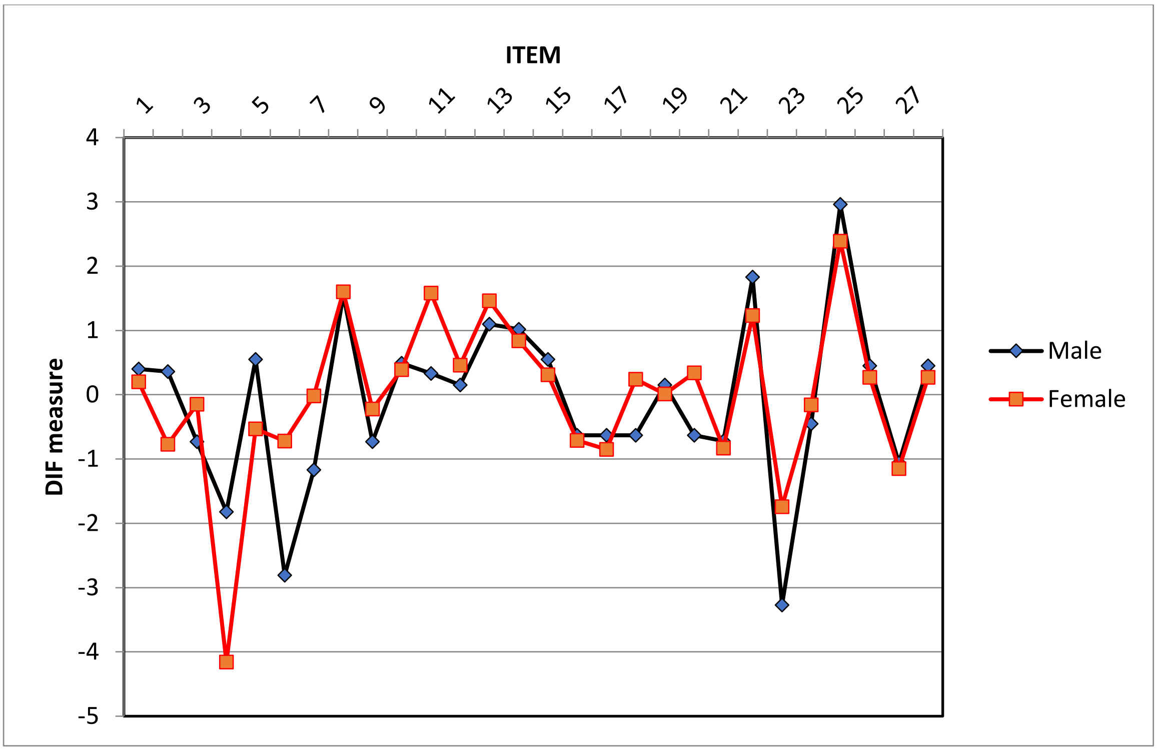


**Supplementary Figure S1**: Differential item function (DIF) measures according to sex for each Impact of Vision Impairment questionnaire item from the Laser intervention in Early Age-related macular Degeneration (LEAD) Study (n = 288).

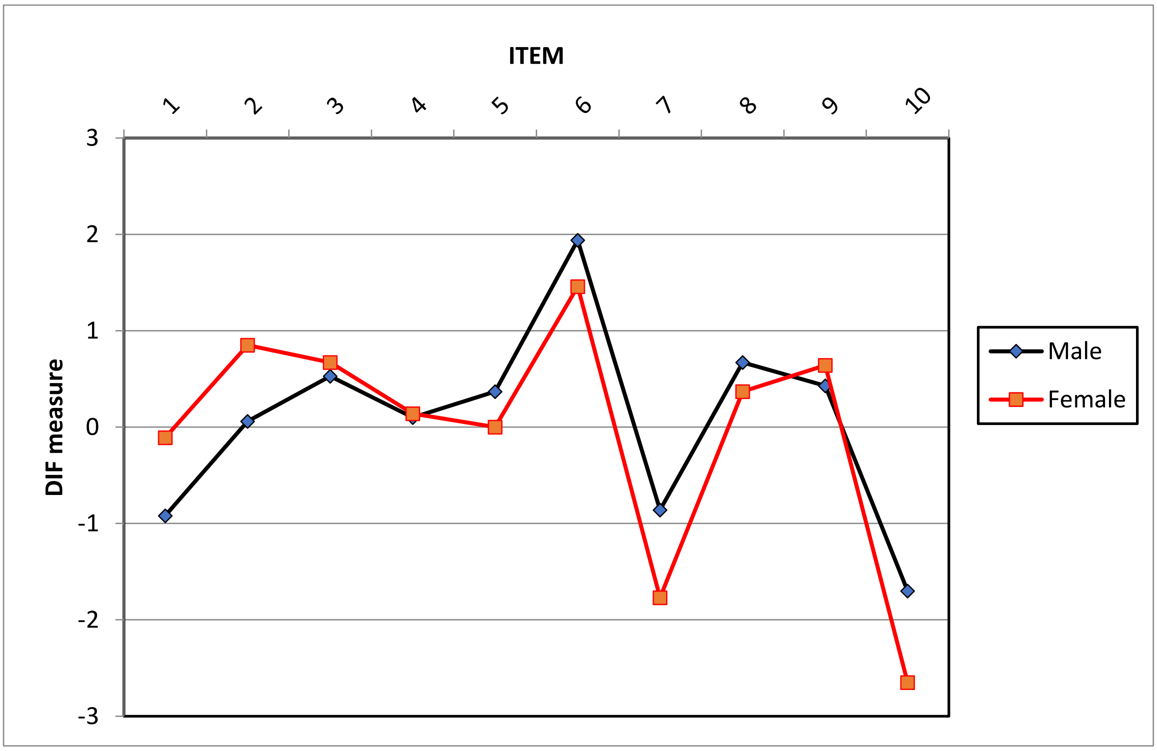


**Supplementary Figure S2**: Differential item function (DIF) measure according to sex for each Night Vision Questionnaire items from the Laser intervention in Early Age-related macular Degeneration (LEAD) Study (n = 288).

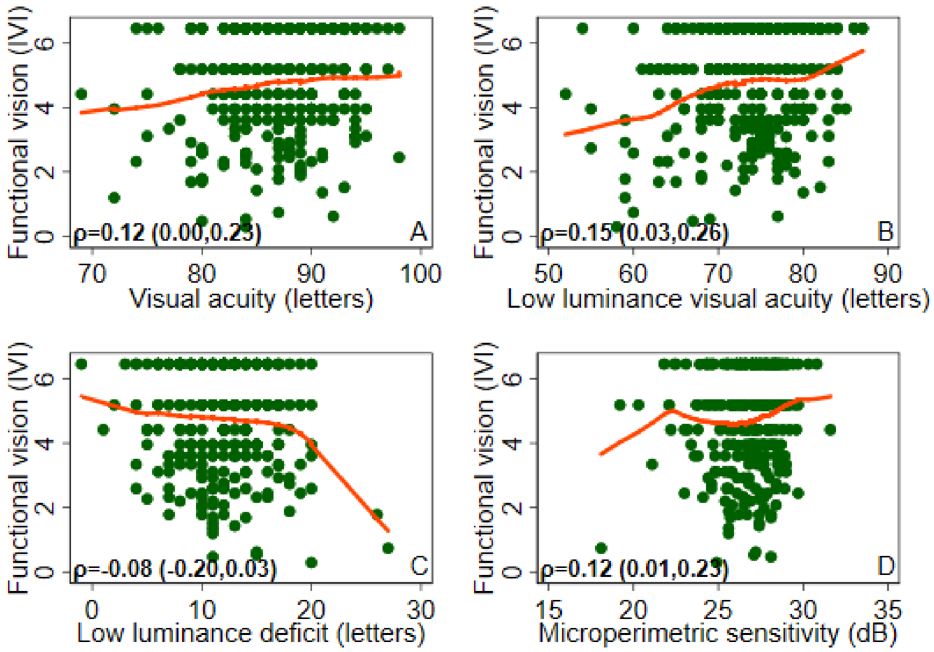


**Supplementary Figure S3**: Scatter plot of measures of visual function at baseline against ability scores generated via a grouped rating scale model of the Impact of Visual Impairment (IVI-28) questionnaire for participants of the Laser intervention in Early stages of Age-related macular Degeneration (LEAD) Study. Measures of visual function are taken from the best performing eye for that measure. Line indicates LOWESS smoother function. $\rho$ is Spearman's correlation coefficient with 95% confidence interval. (A) best corrected visual acuity (B) low luminance visual acuity (C) low luminance deficit (D) microperimetric sensitivity.

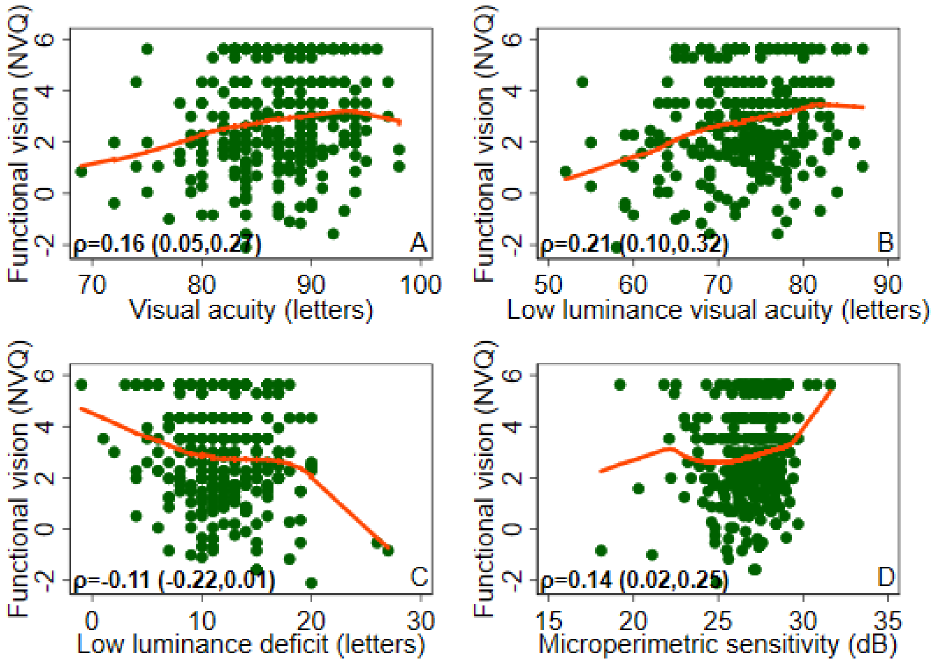


**Supplementary Figure S4:** Scatter plot of measures of visual function at baseline against ability scores generated via a grouped rating scale model of the Night Vision Questionnaire (NVQ-10) for participants of the Laser intervention in Early stages of Age-related macular Degeneration (LEAD) Study. Measures of visual function are taken from the best performing eye for that measure. Line indicates LOWESS smoother function. $\rho$ is Spearman's correlation coefficient with 95% confidence interval. (A) best corrected visual acuity (B) low luminance visual acuity (C) low luminance deficit (D) microperimetric sensitivity.
